# Supplementary material for: Aspirin-Triggered Resolvin D1 Reduces Chronic Dust-Induced Lung Pathology without Altering Susceptibility to Dust-Enhanced Carcinogenesis
Source: Cancers (Basel). 2022 Apr 9;14(8):1900. doi: 10.3390/cancers14081900 (PMC9032113; doi:10.3390/cancers14081900)
Supplement: Supplementary file 1 [file cancers-14-01900-s001.zip › cancers-1641389-supplementary.pdf]

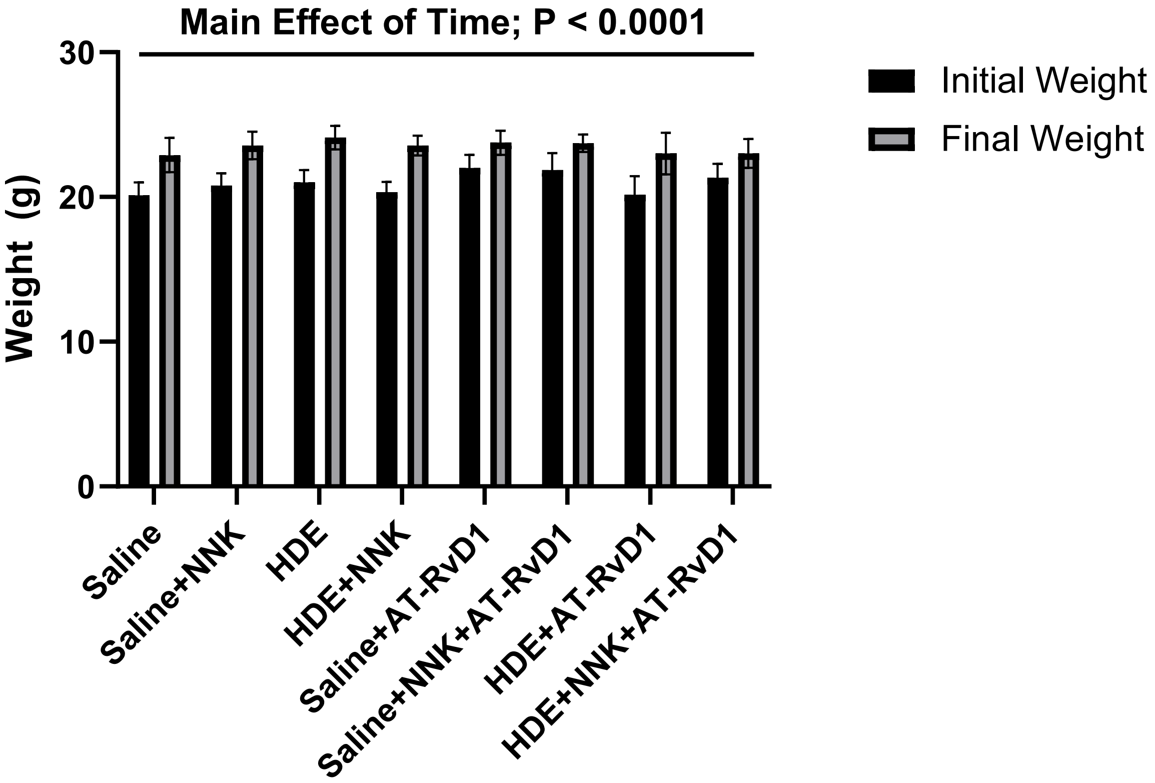

Figure S1. Mice initial and final weights following 24-weeks of chronic exposure. Two-way ANOVA identified a significant main effect of time ($p < 0.0001$) for weight gain in all experimental groups over the 24-week exposure period with HDE, NNK, or AT-RvD1 administration having no impact.

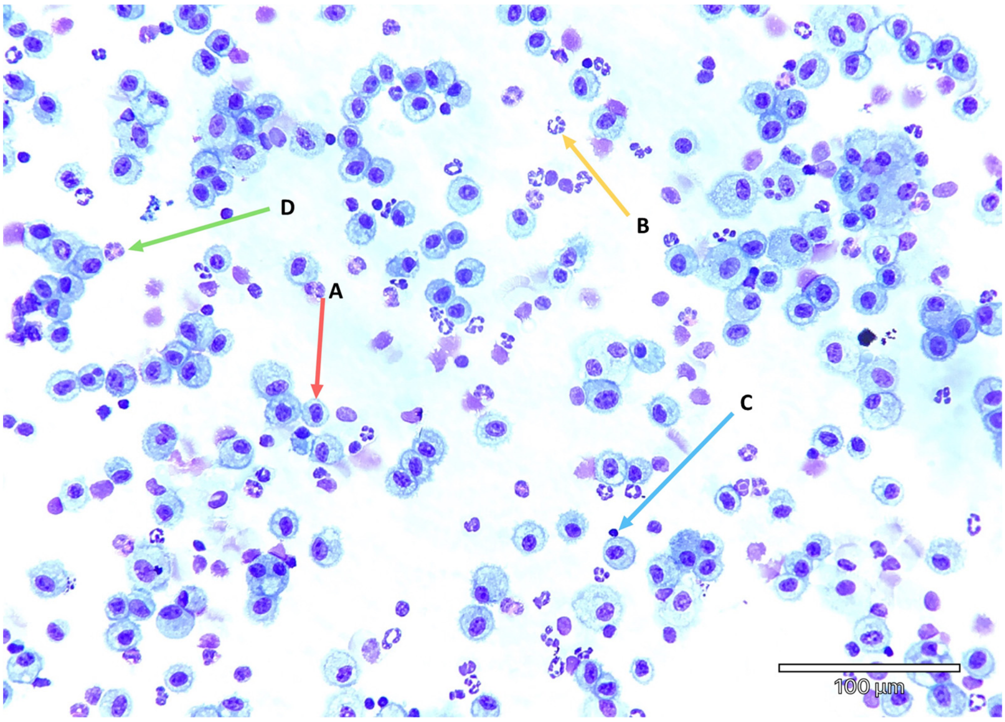

Figure S2. Representative image of BALF immune cell differential analyses. Differential analyses allowed for distinction of immune cells recruited including (A) macrophages, (B) neutrophils, (C) lymphocytes, (D) eosinophils.

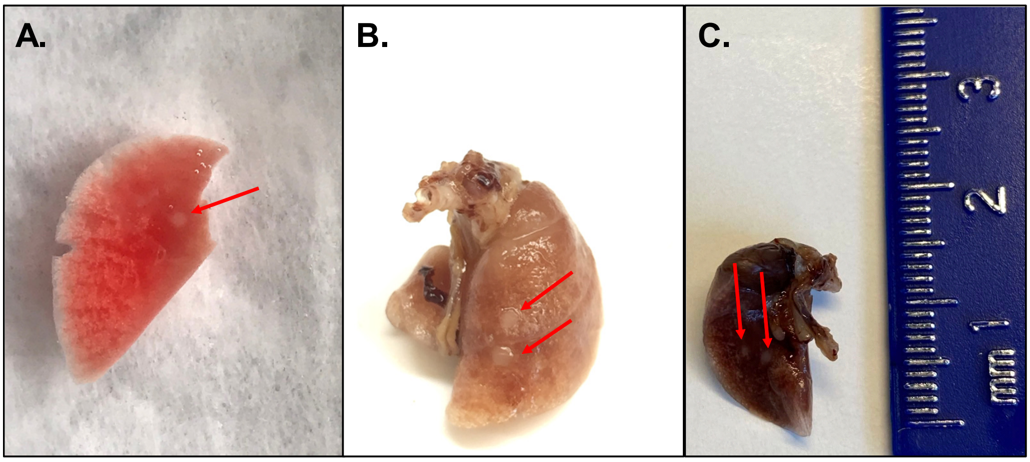

Figure S3. Images of left and right lung tissues with adenomas. (A) Left lung tissue from saline+NNK-exposed mouse that was used for NanoString analyses and oxylipin analysis (B-C) Right HDE+NNK-exposed mouse lung inflated with formalin and used for histopathological analyses. Red arrows point to lung adenomas on the lung tissues.

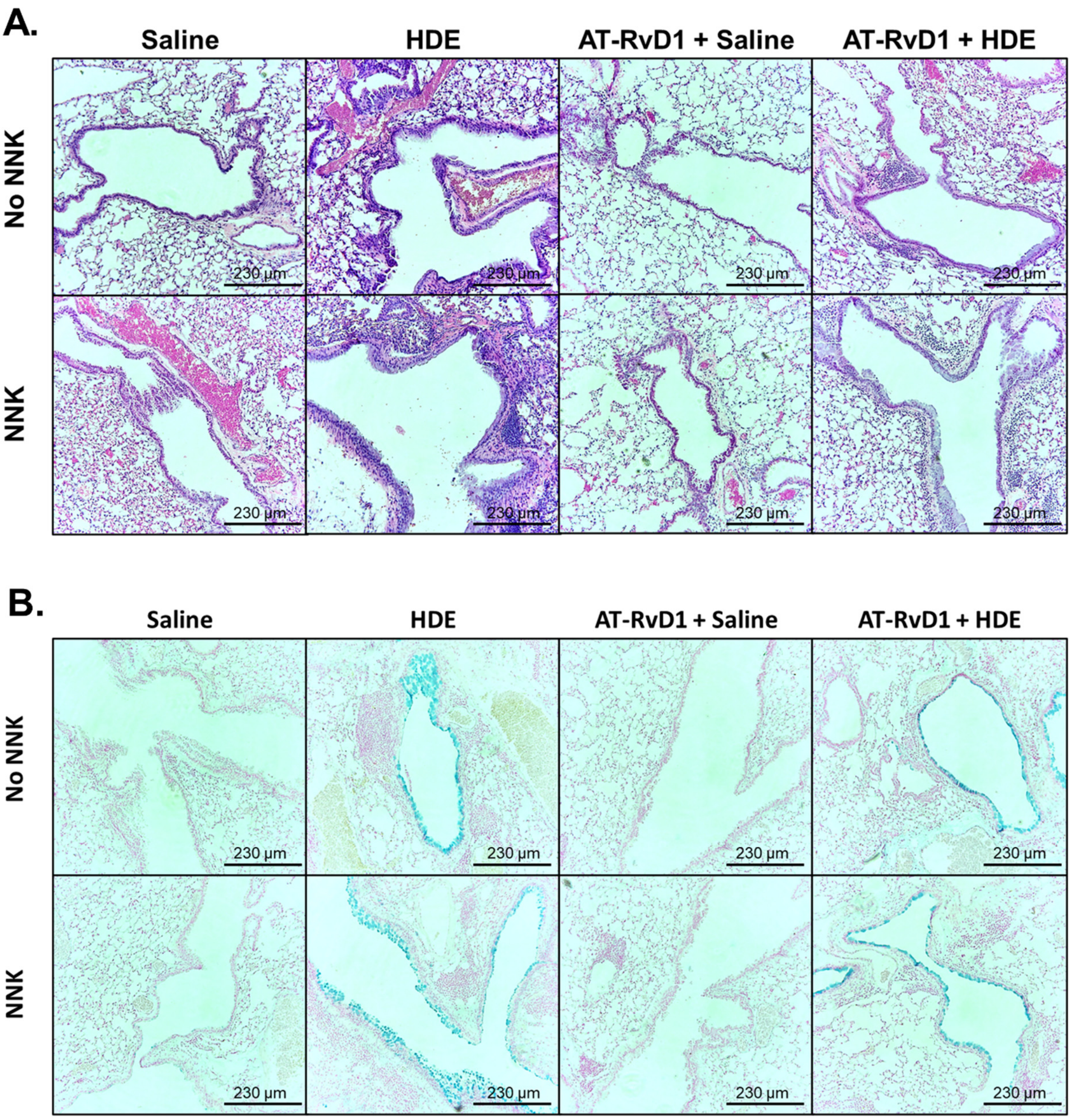

Figure S4. Murine lung tissue histopathology at the conclusion of 24-weeks of chronic HDE exposure. H&E staining was used to assess changes in (A) bronchial/vascular inflammation while Alcian Blue staining was used to assess (B) goblet cell hyperplasia. There was a significant ($p < 0.0001$) main effect of HDE for each of these pathological outcomes. All images were taken using a 10x objective.

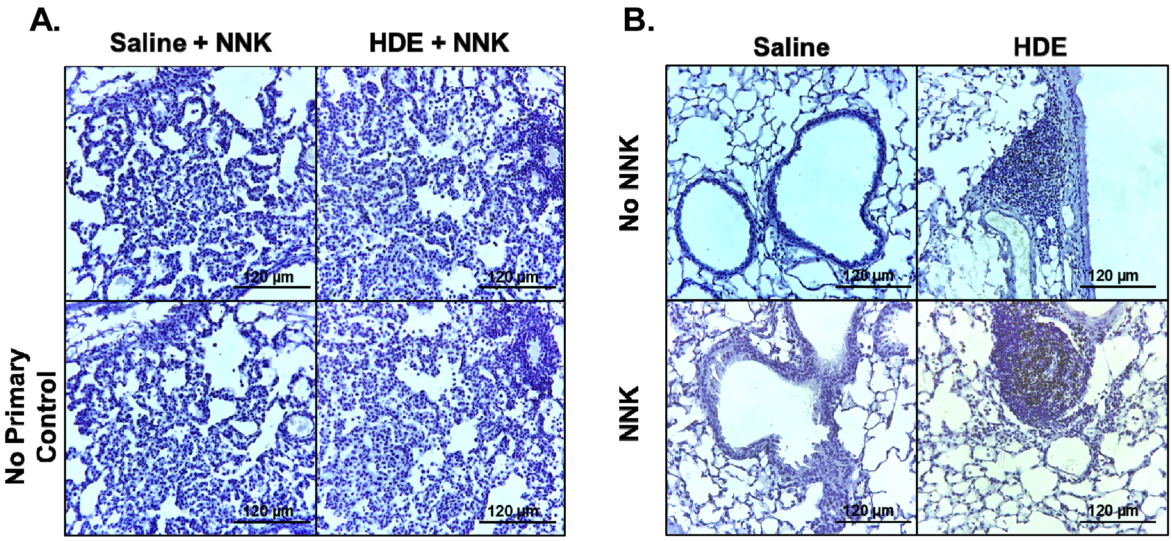

Figure S5. Ki-67 immunohistochemistry staining of FFPE lung tissues. Right lung tissues were formalin-fixed, and paraffin embedded and stained for the cell proliferation marker Ki-67. (A) Staining within lung adenomas of NNK-exposed mice showed Ki-67 expression. (B) Ki-67 expression was prominent in HDE lymphoid aggregates and along the bronchi/ bronchioles of exposed mice.

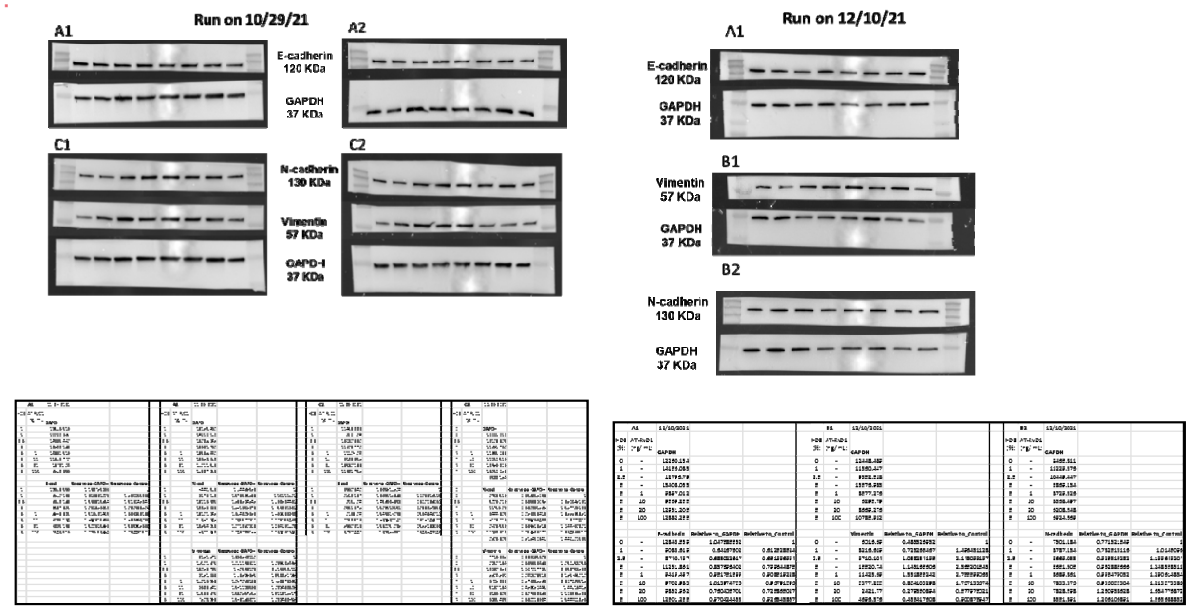

Figure S6. Whole blots (uncropped blots) and densitometry readings/intensity ratios of measured EMT markers. Following treatment with dust, cell lysates were collected from A549 cells and used to measure protein levels of the EMT-related markers E-cadherin (120 KDa), N-cadherin (130 KDa), and vimentin (57 KDa). GAPDH (37 KDa) was used as the loading control.
